# Effects of shutter transients in molecular beam epitaxy

**DOI:** 10.1186/1556-276X-7-620

**Published:** 2012-11-12

**Authors:** Shin-ichiro Gozu, Teruo Mozume, Haruhiko Kuwatsuka, Hiroshi Ishikawa

**Affiliations:** 1 National Institute of Advanced Industrial Science and Technology (AIST), Network Photonics Research Center, AIST Tsukuba Central2, 1-1-1 Umezono, Ibaraki, 305-8568, Tsukuba, Japan

**Keywords:** Molecular beam epitaxy, Shutter transients, X-ray diffraction, X-ray reflectivity

## Abstract

We have studied the effects of shutter transients (STs) in molecular beam epitaxy (MBE). Two series of samples were grown by MBE and evaluated by X-ray diffraction (XRD) and X-ray reflectivity (XRR) measurements. The effects of STs were evaluated by growth rate (GR) analysis using a combination of growth time (GT) and thickness evaluated by XRD and XRR measurements. We revealed two opposite effects of STs: (1) overshoot of GR and (2) increase in GR with GT and subsequent saturation. Each effect was consistent with the previous studies; however, the previous studies showed no relationships between these two effects. By considering closing time of the shutter, the two opposite effects were well understood.

## Background

Molecular beam epitaxy (MBE) is an ideal method to grow nano-structures. MBE allows for the controlled growth of films with sharp doping profiles and different chemical compositions changing over a spatial depth of several angstroms. Multi-layer structures with alternating doping (n, p, or intrinsic) or alternating band gaps can be grown. In addition, self-organized effects can be applied for growing the nano-structures with controlled dimensions that are not only in the growth direction (thickness) but also laterally in the plane of the growth surface. These nano-structures are well known as quantum wells (QWs), quantum wires, and quantum dots (QDs). When their sizes are decreased, the quantum confinement effect becomes the dominant contribution to their electric and optical properties. Because quantum confinement energy is inversely proportional to size (1/L), accuracy of size is very important to fabricate the nano-structures. However, MBE cannot avoid the effects of shutter transients (STs) by which a prominent inaccuracies are caused by strong flux transients after opening the shutter due to temperature gradients in the cells. In an MBE system, beam sources are provided by thermal evaporation of solid source materials in the cells, and the beam sources are individually controlled by mechanical shutters to obtain the intended structures. The mechanical shutters also reflect heat back into the cells. Therefore, a temperature gradient occurs when the mechanical shutters are moved. The studies conducted for STs were contrasting in two ways: (1) growth rate (GR) decreased and was saturated after a characteristic time constant (CTC) [1-4], and (2) the GR was below the desired GR and then approached the target GR within the CTC [5,6]. Therefore, the effects of STs seemed to be contradictory or to have a machine dependence. Therefore, we have studied the effects of STs for our MBE system.

## Methods

### Evaluation method of shutter transients

In order to evaluate STs for MBE, growth time (GT) dependence of GR must be evaluated. Reflection high-energy electron diffraction (RHEED) in MBE is a powerful tool to evaluate GR. During growth, the intensity of oscillation at a specular spot can be observed, and 1 period of the oscillation corresponds to the growth of 1 mono-layer (ML). Evolution of the oscillation can be used to evaluate the GT dependence of the GR. Previous studies have used this method to evaluate STs [2,4,5]. However, GR evaluation using RHEED has some drawbacks. The oscillation is damped. Although substrate rotation is required to obtain good thickness uniformity on the samples, substrate rotation is not possible during RHEED oscillation measurements. This means that only one position in the sample is measured, and thus, some error is involved in this method. In addition, observation of the oscillation is strongly dependent on the growth condition. To overcome these drawbacks, in the present study, some samples were grown, and then the thickness of the samples was evaluated using X-ray diffraction (XRD) and reflectivity (XRR) measurements. XRD measurements can be used to evaluate the layer thickness when the sample has a periodic structure. Because of satellite peak patterns, separation that corresponds to the period can be distinguished. On the other hand, XRR measurements do not require a periodic structure to evaluate thickness. To evaluate the thickness of individual layers by XRR, model-based fitting is required. Because the GT of the samples is known, the GR can be calculated. In this study, InGaAs layers were targeted to evaluate the GT dependence of the GR.

### Experiments

**Figure 1:**
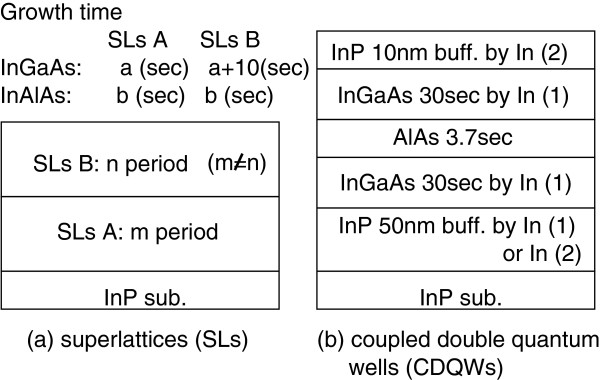
**Schematic structure of the samples. (a)** Superlattice samples with InGaAs growth time ‘a’ and InAlAs growth time ‘b’. **(b)** CDQW samples with growth time for InGaAs and In cells used for this growth.

Our MBE system (VG-V80H) was equipped with solid sources of Al, Ga, As, P, and two sources of In(1,2). Al, Ga, and In(1) were standard VG cells, while In(2) was a dual-filament cell provided by Veeco Instruments, Inc. (St. Paul, USA). As and P were valved cracking cells where the cracking temperatures were 900°C. We grew two series of the samples to consider the properties of both measurements as discussed in the previous section. The first series used sets of two stacked superlattices (SLs) for XRD measurements, and the second series used a set of coupled double quantum wells (CDQWs) for XRR measurements. Each series was grown the same day to minimize fluctuations in the flux of the sources. All samples were grown at 460°C. XRD spectra were taken under the symmetric [004] reflection geometry using a high-resolution XRD system with a four-crystal monochromator. XRR spectra were taken using the same system with a parallel plate collimator. SL and CDQW samples consisted of In_0.4_Al_0.6_As and In_0.72_Ga_0.28_As layers, and In_0.8_Ga_0.2_As and AlAs layers, respectively. The difference in the In content of the InGaAs between the SL and CDQW samples was due to strain compensation condition between the two layers. The schematic structure of the samples is shown in Figure 1. For the SL samples, only In(1) was used. GT for the InGaAs was only varied for 10 s between the two stacked SLs for aiming to evaluate the GR at a fixed GT by subtracting the periods of the stacked two SLs. Growth interruption (GI) between InGaAs and InAlAs was set at 5 s for the SL samples. The number of stacks for the two stacked SLs was different to distinguish each period by taking into account of full width at half maximum (FWHM) of the diffraction peaks. Conversely, GT was the same for the two InGaAs layers of CDQWs. However, two In cells were used individually for a bottom InP buffer of 50 nm and labeled CDQW(1) and CDQW(2) as shown Figure 1. GIs were set at 5 s for the As/As interface and 10 s for As/P interface of the CDQWs, respectively. Because the evaluation of XRR measurements was based on fitting, CDQW samples were evaluated by photoluminescence (PL) measurements to check the accuracy of the evaluation. PL spectra were taken under a 514.5 nm Ar ion excitation laser at 77 K.

## Results and discussion

### Superlattice samples

**Figure 2:**
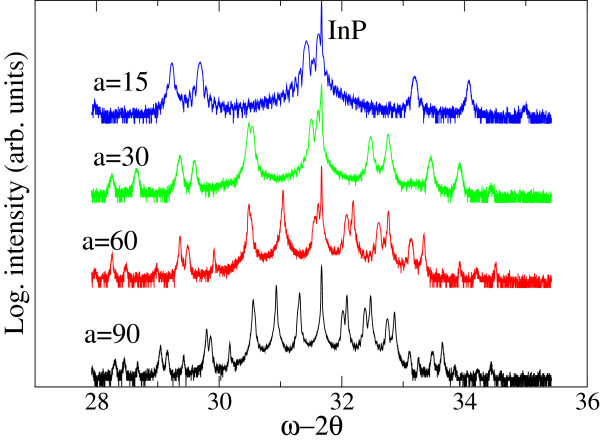
**XRD spectra of the samples.** ‘a’ means InGaAs growth time (s) as shown in Figure 1.

**Figure 3:**
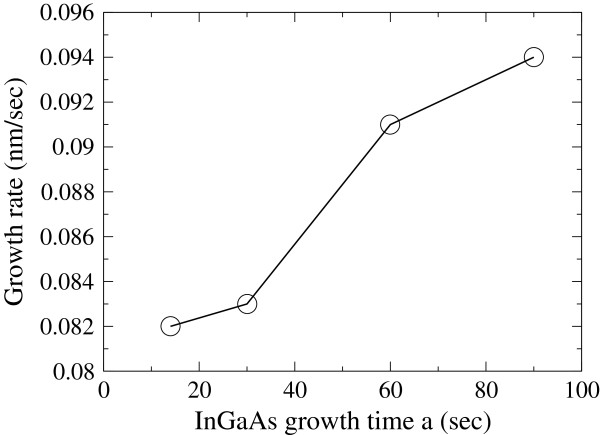
**Growth time dependence of growth rate for InGaAs.** The growth rate is determined by the differences in the SL periods between the two stacked SLs.

XRD spectra of the samples are shown in Figure 2. Sharp and strong peaks at the center of each spectrum correspond to InP substrate. Two sets of the periodic peaks with different FWHMs were distinguished. When the GT of InGaAs was long, the difference in the periods between two SLs was small. This made it difficult to find each of the two peaks in the SLs. However, the difference in periods was clearly distinguishable among the higher satellite peaks. These differences in periods for all samples were used to calculate GR. Figure 3 shows GT dependence of GR for InGaAs. It can be seen that GR was initially low and then increased with GT by a factor of up to 14% during GT testing. Particularly, GR was increased almost 10% when GT was 30 to 60 s. This dependence was consistent with the studies [5,6]. Therefore, the dependence can be understood as the effect of STs. The effect could be attributed as follows: when the shutter was opened, heat radiation from the cell instantly lowered the surface temperature of the source located at inside the cell (Tsurface). Then, Tsurface was increased due to stabilization in the entire cell structure. These changes in Tsurface were responsible for the GT dependence of GR. It should be noted here that the difference in GT of InGaAs was only 10 s between the two stacked SLs. Consequently, estimated GR for shorter GT should have errors.

### Coupled double quantum well samples

**Figure 4:**
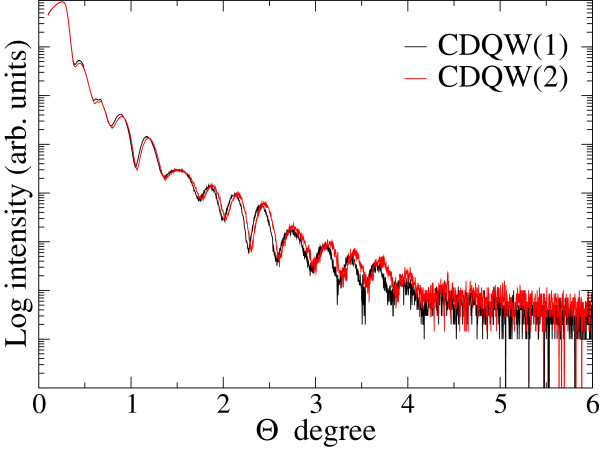
**XRR spectra of CDQW samples.** Black and red lines correspond to spectra of CDQW(1) and CDQW(2), respectively.

**Figure 5:**
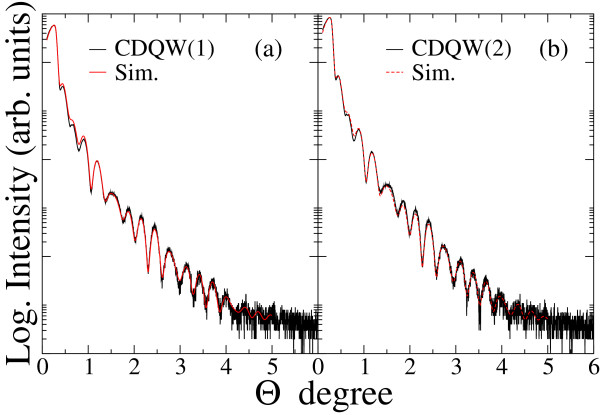
**Experimental XRR spectra of CDQW samples with simulation results for the best fits.** Black and red lines correspond to experimental and simulation spectra, respectively. **(a)** CDQW(1), **(b)** CDQW(2).

**Figure 6:**
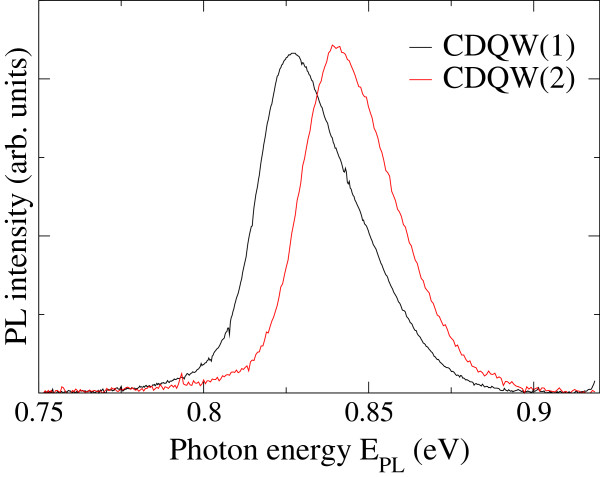
**Photoluminescence spectra of CDQW samples.** Black and red lines correspond to the spectra of CDQW(1) and CDQW(2), respectively.

Figure 4 shows XRR spectra of CDQW samples. Due to the identical structure of the two CDQWs and the same growth condition, these CDQWs should have exhibited identical spectra in XRR measurements. However, the spectra revealed slight periodic differences for higher angles that indicated structural differences between the two CDQW samples. To evaluate their structures, simulations for XRR spectra were performed. Results of comparing the simulations with experimental XRR spectra are shown in Figures 5a,b; the fitting results are listed in Table 1. For the simulations, the model structure was almost the same as the intended structure as shown Figure 1b. The only difference was that a surface oxide layer was added to the model structure. Sample size and X-ray beam width and divergence were also included in the simulation because these parameters were critical due to the glancing angle of the incident X-ray beam in the XRR measurements. The simulation spectra agreed well with the experimental spectra; therefore, the simulations were successful. The simulation results listed in Table 1 show that the thicknesses of the top InP layer and the InGaAs layers were different, although the intended structure was identical between the two CDQWs. Due to the sensitivity of the surface to XRR measurements, the topmost layer was difficult to evaluate [7]. Therefore, the difference in structure was mainly due to the thicknesses of the InGaAs layers. The bottom InGaAs layer was thicker than the upper layers. Due to the same time for growing InGaAs, GR of InGaAs layers exhibited overshoot. This behavior was consistent with the studies [1-4] that revealed flux or GR overshoot. Figure 6 shows PL spectra of the CDQW samples. The spectra revealed the PL peaks were 0.89 and 0.84 eV for CDQW(1) and CDQW(2), respectively. This difference in their peaks was consistent with their structures because the bottom InGaAs layer was thicker for CDQW(2) compared with CDQW(1). The increase in thickness of the bottom InGaAs layer was larger in CDQW(2) sample. The difference in the growth conditions between CDQW(1) and CDQW(2) was the In cell that was used for growing the InP buffer layer. Due to the growth condition, the In(1) and Ga cells were opened first to grow the bottom InGaAs layer of CDQW(2), whereas only the Ga cell was opened first for CDQW(1). Therefore, the flux overshoots of both In(1) and Ga cells occurred to grow InGaAs of CDQW(2), whereas only the Ga cell was under the flux overshoot for growing InGaAs of CDQW(1) that was responsible to the increase in thickness of bottom InGaAs. The flux overshoot could be related with the change in Tsurface. Tsurface was slightly higher than the desired temperature when closing time of the shutters was long, because the shutter reflected the heat back into the cell and because the thermocouple for the cell was located outside of crucible of sources. On the other hand, Tsurface was lowered by the temperature gradient due to the opening of the shutter. These changes in Tsurface for the In and Ga cells could be attributed to the flux overshoot.

**Table 1:** XRR simulation results for the two CDQW samples

**Simulation results**		
**Layer**	**CDQW(1)**	**CDQW(2)**
InP buffer 10 nm	10.8	11.1
InGaAs 30 s	2.90	2.73
AlAs 3.7 s	0.41	0.41
InGaAs 30 s	3.18	3.35
InP buffer + sub.	N/A	N/A

Unit of thickness is expressed in nanometers.

### Consideration of shutter transients

We observed two opposite types of STs in the previous two sections. First was the increase in GR with GT, and second was the overshoot of GR. These behaviors were inconsistent with each other. The difference between the two experiments was the sample structure and the evaluation method. The first was the stacked SLs with XRD measurements, and the second was the single CDQW with XRR measurements. Because XRD measurement of the stacked SLs revealed an average GR for the whole structure, while the XRR measurements of the single CDQW revealed an initial state of growth, the two opposite behaviors could be attributed to long or short shutter closing times. Therefore, the effects of STs in MBE involved two opposite behaviors depending on the shutter closing time. However, Celii et al. [4] reported that STs for their MBE system only showed the overshoot of GR whose amplitude and decay time were related with the shutter closing time. On the other hand, other studies [1-3,5,6] reported one type of STs without the consideration of the shutter closing time. Due to the fact that the effects of STs were strongly dependent on the MBE system, our observations of the two types of STs were characteristics of our MBE system because an amount of the heat back into the cell must depend on the whole structure of the cells. However, the origin of STs arose from the temperature gradient in the cell when the mechanical shutter was moved. This origin is not avoided; therefore, the effects of STs can be said that there is universal phenomena in the MBE system. It should be noted here that our evaluation methods for the effects of STs were different with respect to the previous studies [1-6]. The main difference was that XRR measurement was used to evaluate STs for the initial state of growth. The previous studies used to evaluate STs for their MBE systems as following: beam flux measurements [1], RHEED measurements [2,5,6], optical absorption measurements of QW samples, reflection mass spectroscopy [3], and XRD measurements of SL samples [6]. These measurements as well as XRR measurement are powerful methods to reveal the effects of STs, but each measurement has some drawbacks. The drawbacks for RHEED measurements have already been pointed out in the previous section. However, XRD and XRR measurements which were used in this study have a merit of high accuracy of thickness evaluation. In addition, XRR measurement does not require the periodic structure like SLs which are used for XRD measurement. This feature is very important to reveal the initial state of growth as mentioned before. Therefore, a combination of the evaluation methods used in this study is well balanced and suitable to reveal an entire feature of STs. On the other hand, our experimental results revealed that 5% to 10% of the error in the thickness easily occurred for the designed structure at around 3 nm. Therefore, the effects of STs should be carefully taken into account if the size of the intended structure is as small as a few nanometers.

## Conclusions

We have studied the effects of STs in MBE, and two opposite effects were found. Each effect was consistent with previous studies; however, the previous studies showed no relationships between them. Our experimental results could be categorized into two situations: long and short closing times of the shutters. By categorizing these situations, the two opposite effects were understood. Finally, we pointed out that the effects of STs should be carefully taken into account if the size of the intended structure is as small as a few nanometers.

## Competing interest

The authors declare that they have no competing interests.

## Authors’ contributions

SG carried out the MBE growth, the XRD, XRR, and PL measurements, and wrote the manuscript. TM supported the PL measurements, participated in the analysis of the experimental results, and discussed the results. HK and HI discussed the results. All authors read and approved the final manuscript.

## References

[B1] Miller JN Flux noise in effusion cells: a key to understanding oval defects J Vac Sci Technol B 1992 10 803 10.1116/1.586120

[B2] Heyn Ch Cunis S Shutter transients during solid-source epitaxy J Vac Sci Technol B 2014 25 2005

[B3] Cristea P Fedoryshyn Y Holzman JF Robin F Jäckel H Müller E Faist J Tuning the intersubband absorption in strained AlAsSb/InGaAs quantum wells towards the telecommunications wavelength range J Appl Phys 2006 100 116104 10.1063/1.2400794

[B4] Celii FG Kao YC Beam EA Duncan WM Moise TS Molecular-beam epitaxy flux transient monitoring and correction using in situ reflection mass spectrometry J Vac Sci Technol B 1993 11 1018 10.1116/1.586860

[B5] Roch T Andrews AM Fasching G Benz A Schrenk W Unterrainer K Strasser G High-quality MBE growth of AlxGa1-xAs-based THz quantum cascade lasers Cent Eur J Phys 2007 5 244 10.2478/s11534-007-0004-y

[B6] Andrews AM Roch T Benz A Fasching G Schrenk W Unterrainer K Strasser G Optimization of MBE growth parameters for GaAs-based THz quantum cascade lasers AIP Conf Proc 2007 893 51

[B7] Colombi P Agnihotri DK Asadchikov VE Bontempi E Bowen DK Chang CH Depero LE Farnworth M Fujimoto T Gibaud A Jergel M Krumrey M Lafford TA Lamperti A Ma T Matyi RJ Meduna M Milita S Sakurai K Shabel’nikov L Ulyanenkov A Van der Lee A Wiemer C Reproducibility in X-ray reflectometry: results from the first world-wide round-robin experiment Appl Cryatallography 2008 41 143 10.1107/S0021889807051904

